# Advances in neuropsychiatry: From objective diagnostics to mechanism-based therapeutics

**DOI:** 10.1016/j.neurot.2026.e00854

**Published:** 2026-02-17

**Authors:** Andrew A. Pieper, Francis S. Lee

**Affiliations:** Department of Psychiatry, Case Western Reserve University, Cleveland, OH, 44106, USA; Brain Health Medicines Center, Harrington Discovery Institute, University Hospitals Cleveland Medical Center, Cleveland, OH, 44106, USA; Geriatric Psychiatry, GRECC, Louis Stokes VA Medical Center, Cleveland, OH, 44106, USA; Institute for Transformative Molecular Medicine, School of Medicine, Case Western Reserve University, Cleveland, OH, 44106, USA; Department of Pathology, Case Western Reserve University, Cleveland, OH, 44106, USA; Department of Neurosciences, Case Western Reserve University, Cleveland, OH, 44106, USA; Department of Psychiatry, Weill Cornell Medical College, New York, NY 10065, USA

Recent advances in neuropsychiatry are reshaping diagnosis and treatment across a wide spectrum of disorders. Improved diagnostic methods for delirium, a highly prevalent syndrome, especially in older adults, are enabling faster, more accurate identification and earlier initiation of care that can reduce morbidity and mortality. Refined approaches are improving characterization of psychiatric symptoms in patients with Alzheimer's disease (AD), enabling more personalized interventions. Novel therapies are emerging for the neuropsychiatric complications of human immunodeficiency virus (HIV), while innovative strategies for substance use disorders are expanding harm-reduction and recovery options. Schizophrenia care is entering a new phase with targeted modulation of muscarinic receptors. Behavioral and biological treatments are improving outcomes in autism spectrum disorder (ASD), and multidisciplinary, evidence-based approaches are strengthening care for patients with eating disorders. These advances mark a new era in neuropsychiatry defined by deeper mechanistic insight and more refined, patient-centered treatments, and are reviewed in this special issue of *Neurotherapeutics*.

Yamanshi et al. [1] tackle the challenge of accurately diagnosing delirium, an often-missed, highly morbid complication in hospitalized patients that requires careful, repeated clinical assessment. They describe how bedside electroencephalography (EEG) offers an objective alternative by detecting the characteristic alteration in brain activity that occurs in delirium. While the timing and resources required for conventional EEG makes it suboptimal for routine inpatient use, recently developed portable point-of-care (POC) EEG systems with simplified electrode arrays enable rapid, repeatable bedside recordings by non-specialist staff. Clinical studies show these devices accurately detect delirium, and quantitative bispectral EEG (BSEEG) metrics correlate with delirium severity and can predict adverse outcomes, including mortality, sometimes before clinical signs appear. Complementary rodent studies link EEG slowing and elevated BSEEG to neuroinflammation, microglial activation, and behavioral disruption, supporting a translational link between mechanism and bedside electrophysiology and suggesting value for tracking treatment response. As POC-EEG becomes routine, it could shorten hospital stays, reduce complications, and lower costs. Ongoing work focuses on improving usability, distinguishing delirium-specific from non-delirium EEG patterns, and accounting for individual baseline variability.

Neuropsychiatric symptoms (NPS) in AD, such as apathy, depression, agitation, and psychosis, substantially worsen outcomes for patients and caregivers by reducing quality of life, accelerating decline, increasing caregiver burden, and hastening institutionalization. NPS in AD are frequently under-recognized and undertreated. Rose et al. [2] synthesize evidence linking NPS to network-level physiological dysfunction, neurotransmitter imbalances, neuroinflammation, tau pathology, and circadian disruption. They review pharmacologic options, which generally provide modest benefits with safety concerns, and emphasize effective but underused nonpharmacologic, person-centered strategies such as caregiver training, environmental design, and sensory engagement. The authors highlight emerging technologies, such as neuromodulation, virtual reality, artificial intelligence (AI)-enabled monitoring, and wearable sensors, and propose a paradigm shift toward biomarker-guided behavioral-first care that integrates biological drivers with lived experience, inclusive trial design, and scalable implementation to improve outcomes for patients and caregivers.

As reviewed by Treisman and King [3], psychiatric disorders both drive and result from HIV infection, complicating prevention, diagnosis, adherence, and outcomes. Mental illness is more prevalent among people living with HIV and is linked to higher morbidity, mortality, and transmission risk. Major depression, occurring three-to five-fold more often in people with HIV, exemplifies this bidirectional relationship. It increases vulnerability to acquisition, may arise from viral effects on the brain, and undermines adherence to antiretroviral therapy. Bipolar and personality disorders, traumatic stress, stigma, and social disenfranchisement further amplify risk and disengagement. Substance use disorders are central to HIV transmission dynamics and are under-treated in many HIV clinics. Integrated mental health and addiction services within HIV care consistently improve detection, retention, adherence, and outcomes. Decades of evidence show that sustained investment in comprehensive, accessible psychosocial and psychiatric care is critical to controlling and ultimately eliminating HIV, especially among underserved populations, because biomedical advances such as long-acting agents and preventive tools will only reach their potential when paired with robust behavioral health interventions addressing stigma and social determinants.

A major challenge in treating substance use disorder (SUD) is sustaining long-term abstinence after successfully managing acute withdrawal. Chronic substance exposure alters brain reward circuits, including the endocannabinoid (eCB) system, creating lasting relapse vulnerability. Restoring eCB homeostasis is, therefore, a promising strategy. Directly targeting cannabinoid 1 (CB1) receptors, the principal regulators of the eCB system, risks cannabimimetic side effects and receptor desensitization. Maddox et al. [4] describe an alternative: selectively inhibiting the primary eCB-degrading enzymes fatty acid amide hydrolase (FAAH) and monoacylglycerol lipase (MAGL). These strategies, which elevate brain levels of the eCBs anandamide (AEA) and 2-arachidonoylglycerol (2-AG) and thereby activate CB1 receptors, respectively, promote abstinence by reducing the reward salience of multiple abused substances. Preclinical data show MAGL inhibition has strong potential to reduce opioid relapse, while FAAH inhibitors reduce relapse to psychostimulants such as cocaine. This enzyme-targeting, mechanism-driven approach seeks to restore homeostatic eCB-mediated reward signaling while avoiding the adverse effects of direct CB1 agonists.

Schizophrenia remains a severe, often treatment-resistant disorder whose positive, negative, and cognitive symptoms are only partly addressed by dopamine D2–blocking antipsychotics, which also cause metabolic and motor side effects. Yohn and Paul [5] review emerging therapies that modulate glutamatergic and dopaminergic signaling by targeting M1 and M4 muscarinic acetylcholine receptor (mAchR) subtypes expressed across cortical, striatal, and midbrain circuits. M1 receptors affect cortical glutamatergic processes tied to cognition and negative symptoms, while M4 receptors modulate dopaminergic tone relevant to psychosis. The orthosteric M1/M4-preferring agonist xanomeline provided the first clinical proof-of-concept for mAChR agonism reducing psychosis, and its reformulation with trospium chloride, a peripherally restricted muscarinic antagonist that mitigates peripheral cholinergic adverse effects, has shown robust efficacy and tolerability in Phase 2 and 3 trials and gained regulatory approval. These advances validate mAChR pharmacology as a clinically actionable, non–D2-centric strategy with potential to address the full symptom spectrum. Ongoing efforts aim to develop more selective orthosteric and allosteric M1/M4 modulators, optimize brain penetration and subtype selectivity, and define therapeutic windows, long-term safety, and effects on cognition and function.

Kevelson et al. [6] review emerging therapeutics in ASD across five domains: behavioral and psychosocial interventions, pharmacology, digital/AI tools, neuromodulation, and genetic therapies. They emphasize how the heterogeneity of ASDs complicates research and obscures treatment effects, creating urgent needs for reliable biomarkers, meaningful outcome measures, and equitable access to care and trials. Behavioral therapies remain first-line and are shifting toward naturalistic, developmentally informed, neurodiversity-affirming models that provide adaptive care across the lifespan. Pharmacologic development is moving toward biologically informed, stratified approaches targeting subgroups most likely to benefit. Digital and AI tools promise scalable personalization and access, but raise concerns about validation, regulation, and equity. Neuromodulation and gene-targeted therapies are experimental but advancing. With hundreds of active trials worldwide, progress is rapid. The authors stress the importance of rigorous, inclusive research that integrates mechanistic insight with lived experience to deliver effective, equitable treatments for the diverse autism community.

Lastly, Allam and Attia [7] summarize multidisciplinary advances in treating people with eating disorders, including anorexia nervosa, bulimia nervosa, binge-eating disorder, and avoidant/restrictive food intake disorder (ARFID). Psychotherapy remains central, with family-based approaches showing strong evidence for adolescents with anorexia and bulimia, disorder-specific cognitive behavioral therapy showing efficacy in patients with bulimia and binge-eating disorder, and adapted cognitive behavioral therapy models showing promise for ARFID. Pharmacotherapy is adjunctive, with only two FDA-approved medications: fluoxetine for bulimia nervosa and lisdexamfetamine for binge-eating disorder. Unfortunately, many patients remain nonresponders or relapse. Recent innovations include habit-interruption psychotherapies for anorexia and neuromodulation targeting neural circuits implicated in disordered eating. While promising, these approaches are still only investigational. The authors conclude that progress requires scalable, accessible treatments and integration of biological and behavioral strategies to enhance recovery and prevent recurrence.

The transformative advances covered in this special issue are state-of-art and encouraging. Each piece offers mechanistic insight, clinical implications, and practical takeaways that together chart a path from discovery to bedside impact. These reviews offer evidence, ideas, and interdisciplinary strategies to inform research, improve clinical care, and accelerate the translation of novel diagnostics and therapies that promise better outcomes for patients and families across a broad neuropsychiatric spectrum.

## Author contributions

A.A.P. and F.S.L. conceived and wrote the manuscript and agreed to the published version.

## Declaration of competing interest

The authors declare that they have no known competing financial interests or personal relationships that could have appeared to influence the work reported in this paper.

## Abbreviations

2-AG: 2-arachidonoylglycerol
AD: Alzheimer's disease
AEA: anandamide
AI: artificial intelligence
ARFID: avoidant/restrictive food intake disorder
ASD: autism spectrum disorder
BSEEG: bispectral EEG
CB1: cannabinoid 1
eCB: endocannabinoid
EEG: electroencephalography
FAAH: fatty acid amide hydrolase
HIV: human immunodeficiency virus
mAchR: muscarinic acetylcholine receptor
MAGL: monoacylglycerol lipase
NPS: neuropsychiatric symptoms
POC: point-of-care
SUD: substance use disorder
